# Evaluation of COVID-19 fear and sleep quality in individuals with epilepsy: a cross-sectional study

**DOI:** 10.3389/fneur.2025.1576177

**Published:** 2025-07-02

**Authors:** Hatice Polat, Nuray Bingol, Eda Ay

**Affiliations:** Department of Nursing, Atatürk University, Erzurum, Türkiye

**Keywords:** COVID-19, fear, epilepsy, sleep quality, patient

## Abstract

**Purpose:**

The study was carried out to determine the COVID-19 fear and sleep quality of people with epilepsy.

**Methods:**

The data of this descriptive study were collected from November 2021 to February 2022 with 92 people with epilepsy in the neurology department of a hospital in eastern Türkiye. The Descriptive Information Form, Pittsburgh Sleep Quality Index (PSQI), and COVID-19 Fear Scale were used to collect the data for the study.

**Results:**

The mean COVID-19 score of people with epilepsy was found to be 13.55 ± 7.95, and the mean sleep quality score was 1.67 ± 1.69. Significant differences were detected in the mean fear scores of people with epilepsy according to their working status and perceived health status. It was also found that there was a significant correlation between the duration of epilepsy and sleep quality (*p* < 0.05). No significant differences were detected between the seizure types, number of seizures, number of medications used, presence of comorbid diseases, and fear and sleep quality (*p* > 0.05). No significant relations were detected between the sleep scale and its subcomponents and the COVID-19 Fear Scale (*p* > 0.05).

**Conclusion:**

It was found that the COVID-19 fear levels of people with epilepsy were at a moderate level and their sleep quality was good.

## Introduction

The process that occurred subsequent to the declaration of the pandemic caused an environment of uncertainty for people (1). As the number of patients increased, education was suspended in schools, mass events and sports competitions were postponed, and many public or private businesses were closed temporarily. A quarantine period was made mandatory for people coming from abroad, and their international entries and exits were suspended. Although Türkiye was one of the countries where the virus was transmitted late, the number of patients and deaths continued to increase over time. The total number of cases in Türkiye, which was announced by the Ministry of Health on the COVID-19 Information Platform, was reported to be 17.042.722 in November 2022, and the total number of deaths was reported as 101.492 (2).

It is already known that individuals mostly experience the apprehension of contracting the virus is a prevalent concern. During pandemic periods. The spread of fear also causes anxiety (3), and it is seen that this turns into fear and anxiety at the social level. It is stated that fear, which can be interpreted as an anxiety-based disorder or a psychological reaction, has negative effects on the lives of people because of its consequences (1).

One of the risk groups for COVID-19 is those who have chronic diseases that affect the severity and fatality of COVID-19. As a chronic and neurological disease, epilepsy is the second most prevalent condition after stroke, affecting peoples of all ages (4). Epilepsy prevalence is higher in developing countries compared to developed countries (4). In addition to necessitating extended treatment and regular monitoring, epilepsy leads to disability and imposes socioeconomic burdens (5). Those with epilepsy often experience significant psychosocial issues related to recurring seizures and the fear of social isolation and stigmatization (6).

In terms of COVID-19, individuals who have chronic diseases have some hesitation and fear regarding applying to healthcare institutions to receive health care (7). The risk of a person who has epilepsy contracting COVID-19 is not higher when compared to individuals with other chronic diseases. However, it has been observed that seizures may be triggered by high fever or insomnia because of infection in people with epilepsy. For this reason, such diseases can disrupt seizure control (8).

Research exploring the correlation between epilepsy and COVID-19 with respect to various parameters can be found in the literature. These studies generally focused on medication compliance and stigma (9), psychological effects of COVID-19 (10), anxiety and depression (11), quality of life (12), seizure frequency (13, 14), negative reflections of the pandemic (15) and areas of social life (16).

People with epilepsy may experience sleep problems because of psychosocial problems that originate from the disease (6, 17, 18). Sleep problems are detected in 20–52% of people with epilepsy as an important factor affecting the compliance of people with epilepsy with treatment. However, it has also been reported that the sedation effect of antiseizure medications reduces sleep disruption and awakenings (19). Especially new-generation antiseizure medications cause fewer sleep problems (20, 21). In the literature, studies were detected investigating the correlation between epilepsy and quality of life (17, 18, 22, 23), stress, fatigue, hiding the disease status, and sleep quality of people with epilepsy (24–26). Regular sleep evaluation and the creation of effective solutions are very important in people with epilepsy since they will contribute to seizure control and enhance quality of life.

There is currently a gap in the literature regarding studies on the correlation between COVID-19 fear and sleep quality in epilepsy patients. In this regard, unlike previous psychosocial studies conducted on people with epilepsy, research focusing on the fear of COVID-19 and its impact on sleep quality in people with epilepsy is considered to hold substantial importance for the literature.

## Methods

### Study design

This study was designed using a descriptive and cross-sectional methodology.

### Setting and sample

The study sample comprised individuals diagnosed with epilepsy, who were assessed at the neurology department of a hospital located in eastern Türkiye. *A priori* sample size calculation was performed using G*Power, based on a medium effect size (Cohen’s d = 0.5), a power of 0.80, and an alpha of 0.05, which indicated a minimum required sample of 88 participants. Convenience sampling was used as the sampling method. The research sample consisted of 92 epilepsy patients who met the inclusion criteria and voluntarily participated in the study from November 2021 to February 2022. The inclusion criteria were as follows: being over 18 years of age and having received treatment for epilepsy for a minimum of 1 year, having no other chronic disease, not having been diagnosed with COVID-19 previously, having cognitive competence, not having communication problems. Exclusion criteria for the study were those with chronic/psychiatric diseases other than epilepsy, those diagnosed with COVID-19 or those with active infection, and those with communication or cognitive problems that prevented them from filling out the survey form.

### Ethical consideration

Approval was secured from the Atatürk University Medical Ethics Committee (B.30.2.ATA.0.01.00/32, dated 04.11.2021) before the study began, and written consent was also obtained from the hospital where the study was to be conducted. The authors who conducted the validity and reliability evaluations of the scales used in the study granted their permission for their inclusion. People with epilepsy who met the inclusion criteria and consented to participate were provided with detailed information about the study, and the ‘Informed Consent’ procedure was properly followed. By emphasizing that participation in the study was voluntary, the ‘Respect for Autonomy’ principle was fulfilled. Furthermore, the ‘Confidentiality and Protection of Confidentiality’ principle was ensured by committing to the confidentiality of the participants’ data. The study was carried out in compliance with the principles outlined in the Declaration of Helsinki.

### Measurements/instruments

The Descriptive Information Form, the Pittsburgh Sleep Quality Index (PSQI), the Coronavirus (COVID-19) Fear Scale were used to collect the study data.

#### Descriptive Information Form

The researcher designed a form to collect data on the characteristics of individuals diagnosed with epilepsy. There are questions to determine the age, sex, marital status, employment status, education level, place of residence, other disease, treatment status of people with epilepsy.

#### Fear of COVID-19 Scale (FCV-19S)

The Turkish version of the scale was validated and tested for reliability by Artan et al. (27). The scale has 1 single dimension and 7 items. No reverse items are used in it. The total score reflects individuals’ level of fear concerning the COVID-19. The scores vary between 7 and 35. There are questions such as “I am most afraid of COVID-19”, “Thinking about COVID-19 makes me uncomfortable”, and “When I think of COVID-19, my hands shake” on the scale in which fear, anxiety, and symptoms related to coronavirus are evaluated with a 5-point Likert design. A high score on the scale means having a high level of fear of Coronavirus. The internal consistency of the original scale, as measured by Cronbach’s Alpha, was 0.82, and that of the Turkish version was found to be 0.867. The Cronbach’s Alpha value in this study was calculated as 0.884.

#### Pittsburgh Sleep Quality Index (PSQI)

Developed by Buysse et al. (28) in 1989, assesses sleep quality over the past month. Ağargün et al. (29) conducted the validity and reliability study of the scale in 1996 in Türkiye. The internal consistency, as measured by Cronbach’s Alpha, was found to be 0.80. The scale consists of 24 items, including 19 self-report questions completed by individuals with epilepsy and 5 questions answered by a spouse or roommate. Question 19, which is a self-report question about whether there is a roommate or spouse, is not used in the scoring of the scale. The 18 items used for scoring are categorized into 7 components: “Subjective Sleep Quality, Sleep Latency, Sleep Duration, Habitual Sleep Efficiency, Sleep Disturbance, Use of Sleeping Pills, and Daytime Dysfunction”. Each item is scored on a scale from “0” to “3,” and the sum of the 7 components provides the total PSQI score, ranging from 0 to 21. Scores higher than 5 reflect poor sleep quality. In the present study, the Cronbach’s Alpha coefficient was calculated as 0.67.

### Data collection/procedure

Data collection was performed through in-person interviews with individuals with epilepsy at the Neurology Clinic. On average, it took 10 min to respond to the questions.

### Data analysis

The data were analyzed using nonparametric tests in SPSS version 20 software (IBM/SPSS, Chicago, IL, United States) program. Percentage, arithmetic mean, standard deviation, Pearson Correlation Analysis, Mann Whitney U, Kruskal Wallis, Tukey test, Cronbach Alpha were used in data assessment.

## Results

The mean COVID-19 Fear scores of the people with epilepsy was found to be 13.55 ± 7 0.95, and the PSQI total score mean was 1.67 ± 1.69 (Table 1).

**Table 1:** COVİD-19 fear and PSQI total scores (*n* = 92).

SCALE	Mean ± SD	Minimum–Maximum
COVID-19 fear scale	13.55 ± 7.95	7-35
PSQI total	1.67 ± 1.69	0-10
Sleep quality	0.04 ± 0.20	0-1
Sleep latency	0.48 ± 0.78	0-3
Sleep duration	0.25 ± 0.52	0-3
Habitual sleep efficiency	0.42 ± 0.77	0-3
Sleep disturbance	0.36 ± 0.50	0-2
Use of sleeping medication	0.87 ± 0.48	0-3
Daytime dysfunction	0.03 ± 0.31	0-3

SD, Standard deviation; PSQI, Pittsburgh Sleep Quality Index.

A total of 41 participants (44.6%) were aged between 18 and 27 years, 50 (54.3%) were women, 48 (52.2%) were single, 54 (58.7%) did not have children, 26 (28.3%) were literate, 72 (78.3%) were unemployed, 77 (83.7%) lived in the city. Also, 39 (42.4%) of people with epilepsy had been diagnosed with epilepsy for more than 10 years, 54 (58.7%) had seizures under control, 68.5% had generalized seizure type, 62 (67.4%) were using medication, and 86 (93.5%) did not have any comorbidities (Table 2).

**Table 2:** Descriptive characteristics of individuals with epilepsy and a comparison with COVİD-19 fear and PSQI total score and mean (*n* = 92).

Descriptive characteristics	%	n	COVID-19 fear	PSQI
			Mean ± SD	Mean ± SD
Age (34.9 ± 15.68)				
18–27	44.6	41	13.49 ± 7.29	1.66 ± 1.62
28–37	21.7	20	14.35 ± 8.72	1.40 ± 1.19
38 and over	33.7	31	14.74 ± 8.82	1.87 ± 2.05
Test (*p*)			KW:0.382 (0.820)	KW:0.348 (0.840)
Gender				
Male	45.7	42	12.59 ± 8.19	1.88 ± 2.05
Female	54.3	50	15.32 ± 7.85	1.50 ± 1.31
Test (*p*)			MWU:818.0 (0.053)	MWU:988.0 (0.620)
Marital status				
Single	52.2	48	13.68 ± 7.54	1.87 ± 1.95
Married	47.8	44	14.50 ± 8.68	1.45 ± 1.33
Test (*p*)			MWU:992.0 (0.594)	MWU:957.0 (0.427)
Educational status				
Illiterate	9.8	9	18.11 ± 9.63	1.88 ± 1.45
Literate	28.3	26	14.88 ± 9.67	1.96 ± 2.35
Primer education	21.7	20	13.30 ± 7.46	1.45 ± 1.43
High school	17.4	16	12.62 ± 7.77	1.12 ± 1.02
University and higher	22.8	21	13.19 ± 5.74	1.85 ± 1.42
Test (*p*)			KW:2.367 (0.669)	KW:3.030 (0.553)
Employment status				
Employed	21.7	20	9.65 ± 6.85	1.70 ± 2.55
Unemployed	78.3	72	15.30 ± 8.0	1.66 ± 1.38
Test (*p*)			MWU:417.0 (0.002)*	MWU:599.5 (0.241)
Place of residence				
City	83.7	77	13.83 ± 7.95	1.61 ± 1.75
Town	16.3	15	15.33 ± 8.86	2.00 ± 1.30
Test (*p*)			MWU:509.5 (0.444)	MWU:450.5 (0.168)
Duration of epilepsy (mean:13.60±11.50)				
1-5 years^a^	18.5	17	14.52 ± 7.25	1.76 ± 1.09
6-10 years^b^	39.1	36	15.02 ± 9.02	1.22 ± 1.56
11 and more^c^	42.4	39	13.00 ± 7.54	2.05 ± 1.93
Test (*p*)			KW:1.460 (0.482)	KW:7.224 (0.027)* c>b
Number of Seizure				
1≥ per year	25.0	23	16.00 ± 8.61	1.95 ± 0.97
1≥ per month	9.8	9	12.77 ± 8.19	0.88 ± 0.92
1≥ per week	6.5	6	7.00 ± 0.00	3.00 ± 3.57
Under control	58.7	54	14.25 ± 7.96	1.53 ± 1.67
Test (*p*)			KW:7.095 (0.069)	KW:7.102 (0.069)
Seizure type				
Generalized onset (tonic-clonic)	68.5	63	14.90 ± 8.64	1.68 ± 1.64
Focal onset (Simple and Complex partial)	28.3	26	12.38 ± 6.49	1.69 ± 1.87
Unknown onset	3.2	3	11.33 ± 7.50	1.33 ± 1.52
Test (*p*)			KW:1.538 (0.464)	KW:0.244 (0.885)
Number of drugs used				
Monotherapy	67.4	62	13.87 ± 7.56	1.43 ± 1.63
Polytherapy	28.3	26	15.15 ± 9.43	2.23 ± 1.70
None	4.3	4	10.25 ± 6.50	1.75 ± 2.06
Test (*p*)			KW:1.087 (0.581)	KW:5.693 (0.058)
Presence of a comorbid disease				
Yes	6.5	6	20.83 ± 11.60	1.83 ± 1.60
No	93.5	86	13.60 ± 7.64	1.66 ± 1.70
Test (*p*)			MWU:156.5 (0.087)	MWU:229.0 (0.638)

PSQI, Pittsburgh Sleep Quality Index; SD, Standard deviation; MWU, Mann Whitney U; KW, Kruskal Wallis.

*Bold values mean *p* < 0.05.

a, b, c indicate the ranking of the variables based on post-hoc analysis results.

There were no significant differences observed in the mean scores of COVID-19 fear and sleep quality based on age, sex, marital status, educational background, or place of residence among people with epilepsy (*p* > 0.05). There was a statistically significant difference in the mean COVID-19 fear scores based on working status (*p* < 0.05). The unemployed group has higher COVID-19 fear scores. Also, there was a significant difference in sleep quality based on disease duration (*p* < 0.05). According to the post-hoc analysis, the “11 and more” group accounted for this significant difference. The average score of this group was > the “6–10 years” group. No significant differences were found between seizure type, seizure frequency, number of medications, comorbidities, and the fear of COVID-19 or sleep quality in people with epilepsy (*p* > 0.05) (Table 2). No significant relations were detected between the sleep scale and its subcomponents and the COVID-19 Fear Scale (*p* > 0.05) (Table 3).

**Table 3:** PSQI and fear of COVİD-19 correlation (*n* = 92).

PSQI	Fear of COVID-19	
	r	*p*
Sleep quality	0.025	*p* = 0.817
Sleep latency	0.003	*p* = 0.978
Sleep duration	0.003	*p* = 0.976
Habitual sleep efficiency	0.197	*p* = 0.060
Sleep disturbance	−0.058	*p* = 0.583
Use of sleeping medication	0.057	*p* = 0.587
Daytime dysfunction	−0.092	*p* = 0.381
PSQI Total	0.077	*p* = 0.463

PSQI, Pittsburgh Sleep Quality Index; r, Pearson correlation.

## Discussion

While psychosocial studies on epilepsy have been previously conducted, this study approaches the fear of COVID-19 and sleep quality in the context of current literature findings. The average score on the COVID-19 Fear Scale for people with epilepsy was 13.55 ± 7.95. The scale yields scores ranging from 7 to 35, with higher scores indicating a greater level of COVID-19 fear. The people with epilepsy had moderate fear of COVID-19 in the present study. A study conducted on individuals with Multiple Sclerosis, a neurological illness, indicated mental stress related to COVID-19, while 22% of individuals with epilepsy exhibited moderate or severe anxiety (30). The literature shows that people with epilepsy are vulnerable to special disasters such as pandemics and many studies suggest that they are exposed to psychological stress in many aspects (31). People with epilepsy may be exposed to stressors related to the disease process, especially the inability to obtain/secure antiepileptic medications or to control seizures. Several studies have emphasized the importance of individuals with epilepsy maintaining access to healthcare providers and adhering to antiseizure medications regimens (11, 32). In Türkiye, reporting periods were extended with legal regulations, and antiseizure medications were easily obtained from pharmacies to secure the supply of medicines for individuals with chronic diseases such as epilepsy.

The total mean score was found to be 1.67 ± 1.69 in the PSQI. According to the sleep quality scale used in this study, scores of 5 and above indicate poor sleep quality. Therefore, it can be said that the sleep quality of the epileptic individuals participating in this study was good. Panahi et al. (33) found symptoms of poor sleep quality in a study examining the mental status of individuals with epilepsy during the Covid-19 pandemic. In a surprising finding, people with epilepsy were found to have satisfactory sleep quality in the current study. Similar to our study findings, Uçan Tokuç et al. (34) found that only 30.3% of patients experienced insomnia during the pandemic period. Unlike our study findings, it has been reported in the literature that sleep problems are 2–3 times more common in people with epilepsy than general population, thus limiting the ability of individuals to carry out daily activities effectively (22, 23). In a meta-analysis conducted by Kuroda and Kubota (10), 30–40% of the individuals who had epilepsy reported sleep disorders. The fact that the participants were young (34.9 ± 15.68) may be a factor in good sleep quality in the present study. Age is a factor that affects sleep requirements. As we age, sleep quality deteriorates and the frequency of sleep problems increases (35). However, it is accepted that antiseizure medications normalize sleep by suppressing micro-arousals and reducing sleep fragmentation and awakening with their sleep-stabilizing and sedative effects (19). Having healthy sleep in people with epilepsy not only contributes to seizure control but also contributes to improving the quality of daily life of such individuals (36). Despite the ongoing debate in the literature, some ASMs and epilepsy surgery have been suggested to enhance sleep, likely due to their role in seizure regulation (37). It has been reported that frequent seizures increase the symptoms of sleep disorders in individuals with epilepsy (17). While sleep state has significant effects on epilepsy, the opposite is also true, considering that epilepsy and the presence of antiseizure medications also affect the sleep of people with epilepsy (38).

Individuals with epilepsy who were not employed reported higher levels of fear regarding COVID-19 compared to those who were employed. In a study by Yin et al. (39), it was reported that epilepsy patients with low income level had higher fear of COVID-19. This result suggests that working individuals may experience less fear of COVID-19 because of their interaction with their immediate environment and their work engagement. Working individuals’ maintaining contact with their social environment and continuing their routines may support their ability to cope with the pandemic. The literature also reports that social isolation and low levels of socialization increase feelings of anxiety and fear related to COVID-19 (40, 41).

It was also found in the present study that the sleep quality of people with epilepsy with a disease duration of 11 years or more was lower than other groups. In previous studies conducted on the same subject, no significant correlation was reported between disease duration and sleep quality (17, 25). Duran et al. (42) determined in their study that the sleep quality of individuals with epilepsy was poor, but there was no significant difference between the duration of the disease. It is suggested that the younger mean age of the participants in this study may have contributed to this result.

No correlation was detected in the present study between sleep quality and fear of COVID-19. Contrary to our study finding, a previous study reported that the restrictions during the COVID-19 pandemic could have negatively affected sleep quality with the increased prevalence of psychological disorders (43). In another study, it was reported that worsening seizures in of people with epilepsy might be associated with the difficulty in reaching doctors and medications that may occur in the early days of the pandemic, but as a result of the study, it was determined that there was no disruption in the antiseizure medications treatments of people with epilepsy during the pandemic period (14). Panahi et al. (33) found a relationship between mental health disorders and poorer sleep quality in their studies conducted with individuals with epilepsy during the pandemic period. Interestingly, Gargiulo et al. (44) found that pandemic-related stress was not significantly associated with sleep quality. It is sufficient for people with epilepsy to take the same precautions as everyone else to protect against COVID-19. Medicines used for epilepsy do not cause susceptibility to infections or weaken the immune system. For this reason, it was considered that the anxiety levels and sleep quality of people with epilepsy were not affected significantly. Factors such as the level of social support participants experienced during the pandemic, their access to information, and individual coping strategies may affect the relationship between sleep quality and fear of COVID-19.

### Limitations of the study

Conducting the present study in one single center and generalizing the results only to the region where the study was conducted constitutes the limitations of the study.

## Conclusion

Although people with epilepsy experienced moderate fear of COVID-19, it was found that their sleep quality was not affected by the fear of COVID-19. People with epilepsy whose diagnosis lasted longer had better sleep quality, and people with epilepsy who were not working had a higher COVID-19-related fear.

## Data Availability

The data analyzed in this study is subject to the following licenses/restrictions: can be shared upon request from the authors. Requests to access these datasets should be directed to HP, haticeduyarpolat@gmail.com.
